# A Case of Cytomegalovirus Infection With Severe Thrombocytopenia in an Infant Who Was Successfully Managed With Antiviral Therapy

**DOI:** 10.7759/cureus.30640

**Published:** 2022-10-24

**Authors:** Nadia Echcharii, Nabila Chekhlabi, Nezha Dini

**Affiliations:** 1 Pediatrics, Cheikh Khalifa International University Hospital, Mohammed VI University of Health Sciences, Casablanca, MAR

**Keywords:** cytomegalovirus, infant, ganciclovir, cytomegalovirus-cmv, antiviral therapy, immune thrombocytopenic purpura (itp)

## Abstract

Cytomegalovirus (CMV) is a member of the *Herpesviridae* family of DNA viruses. It is one of the major infectious causes that induce thrombocytopenia. We herein report a case of CMV infection in an infant complicated with severe thrombocytopenia that was successfully managed by antiviral treatment. A three-month-old girl presented with generalized petechial lesions in the limbs, trunk, and eyelids, discovered by chance during a vaccination consultation in an apyretic context.

Blood examinations demonstrated thrombocytopenia at 26,000/mm^3^. She was diagnosed with immune thrombocytopenic purpura. Intravenous immunoglobulin was administered thrice and corticosteroid therapy at a dose of 2 mg/kg/day was started. The evolution during her hospitalization was marked by the increase to 373,000/mm^3^. A week later, the platelet had decreased again to 8000/mm^3^. Positive serology and high CMV DNA detected in serum by real-time quantitative polymerase chain reaction confirmed the diagnosis of CMV infection. In consideration of the severe thrombocytopenia, antiviral therapy with ganciclovir 5 mg/kg/12 hours was initiated. The platelet counts increased with decreasing CMV loads. She was discharged home after clinical stabilization with a close follow-up over one year.

## Introduction

Cytomegalovirus (CMV) is a DNA virus responsible for most often unnoticed infections. Its pathogenic character occurs especially in patients whose immune defenses have been weakened, such as those treated with immunosuppressants, fetuses, and small infants. This group of viruses is capable of causing latent infection with secondary reactivation. Infection with CMV does not confer immunity because reinfection with a new strain is possible. It is only found in humans and is present in all bodily secretions [1]. It is one of the major infectious causes that induce thrombocytopenia. Minimal data are available on the association between CMV infection and thrombocytopenia during early infancy [2]. The most common form of CMV infection is asymptomatic or benign in immunocompetent children [3], but CMV infections occurring during the neonatal period can be serious or even fatal. It is often difficult to differentiate between congenital and acquired CMV infections in newborns.

Usually, antiviral treatment is indicated for severe forms of thrombocytopenia associated with CMV infection as well as refractory forms after treatment with intravenous human immunoglobulins and in premature newborns [3]. However, there is no consensus determining the indication of antiviral therapy [4].

We describe an infant case of CMV infection with severe thrombocytopenia that was successfully managed with antiviral treatment.

## Case presentation

The patient was a three-month-old girl, who presented with generalized petechial lesions discovered incidentally during a vaccination consultation in the context of apyrexia and good general condition. She was born at term with a weight, height, and head size appropriate to her gestational age.

The pregnancy was complicated by gestational diabetes and the discovery of unexplored maternal thrombocytopenia at the end of the third trimester. She was breastfed after birth.

Physical examination showed an afebrile infant who was conscious, responsive, and hemodynamically stable. No lymphadenopathy or hepatosplenomegaly was evident on palpation. We noted the presence of diffuse petechiae on the four limbs, on the trunk, face, and eyelids. There were also bruises of about 1 cm on the lower limbs, and palatal hemorrhagic bubbles without gingivorrhagia or gingival hypertrophy.

Blood tests revealed thrombocytopenia (platelet count: 22,000/ml), non-hemolytic anemia at 10 g/dl, and leukocytosis (white blood cell (WBC) count: 9,870/ml). Other laboratory findings, such as a coagulation test and blood chemistry analysis, were within normal ranges.

The bone marrow showed a marrow rich; all the lines were well represented without excess blasts. An etiological assessment made of serology of CMV, Epstein-Barr virus (EBV), viral hepatitis B and C, parvovirus B19, and antiplatelet antibodies was carried out. The most likely diagnosis was idiopathic thrombocytopenic purpura (ITP) and intravenous immunoglobulin therapy at a dose of 1 g/kg for two days was administered. The evolution was marked by a rapid increase in platelets to 373,000/ml. One week later, the infant had a clinical recurrence of the hemorrhagic syndrome and a drop in platelets to 8000/ml. Despite the second course of IV immunoglobulins followed by corticosteroid therapy at a dose of 2 mg/kg/day, her platelet count remained low with a maximum of 20,000/ml.

The result of infection workups showed that CMV-specific serum immunoglobulin M (IgM) and immunoglobulin G (IgG) antibodies were positive and a large quantity of CMV DNA (73,000 copies/ml) was detected in serum by real-time polymerase chain reaction (PCR), suggesting the acute phase of CMV infection. The maternal CMV serology showed positive CMV IgG and negative CMV IgM, indicating past CMV infection. Congenital CMV infection was excluded because none of the workups revealed any sign of congenital CMV infection: normal brain scan, electroencephalogram, auditory brainstem response, and ophthalmological examination. Given the severity of the disease, an immune assessment relating to the dosage of immunoglobulins, lymphocyte subpopulations, and HIV serology was able to rule out primary or secondary immunodeficiencies.

**Figure 1:**
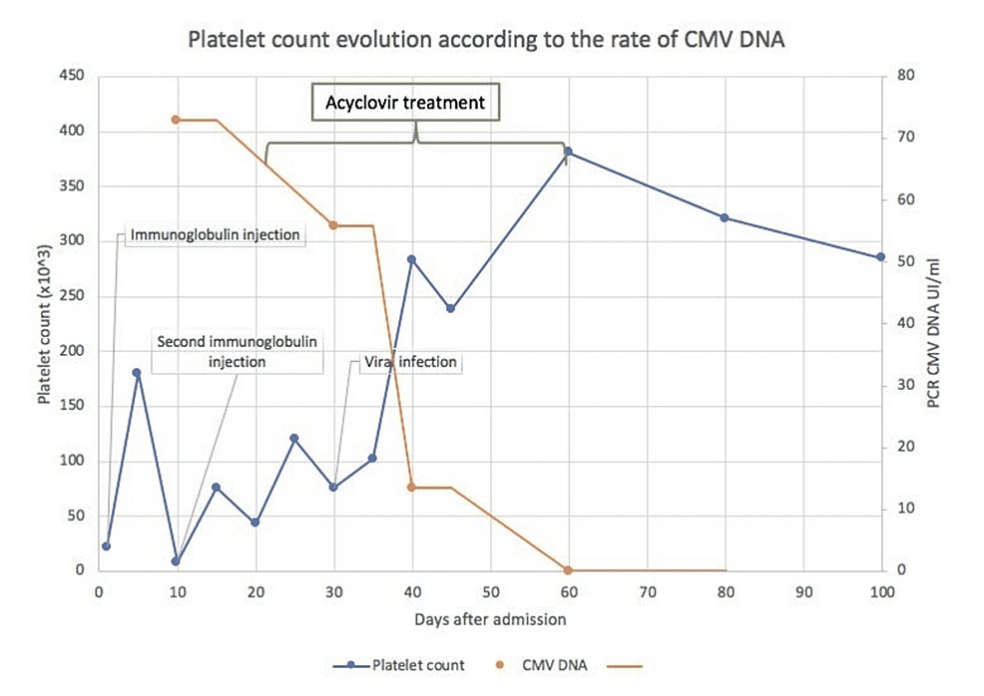
Infant’s clinical course after admission. Platelet count recovered and CMV load decreased after initiating ganciclovir (GCV). CMV: cytomegalovirus; PCR: polymerase chain reaction.

Due to this severe picture and the high CMV viral loads, empiric antiviral therapy with ganciclovir (GCV) 6 mg/kg/12 hours was indicated. After the initiation of GCV, the platelet counts gradually increased with decreasing CMV viral load (Figure 1). After four weeks of antiviral therapy, CMV DNA became undetectable and platelet counts remained within the normal range.

## Discussion

CMV is one of the most common agents that trigger ITP, and it is estimated that CMV may account for 26.4-31.7% of pediatric ITP cases [2,5]. CMV infection is characterized by vertical transmission from mother to infant via perinatal infection or infected breast milk [6].

After three weeks of life, it is difficult to differentiate between congenitally acquired CMV infection and perinatal transmission in infants, unless the clinical features of the former such as chorioretinitis, intracranial calcification, microcephaly, and hearing loss are present [6]. Our patient was explored and a congenital infection was excluded.

The pathophysiology and causality between CMV infection and thrombocytopenia remain uncertain [7,8]. Several suggestions have been put forward, but only two hypotheses have been retained [3,8], i.e., CMV has a direct cytopathic effect on megakaryocytes or CMV induces immunological disorders, such as the formation of antibodies against the platelet membrane [2].

We described an infant case of acquired CMV infection complicated by severe thrombocytopenia, which was treated with antiviral therapy. The consensus of the International Congenital Cytomegalovirus Recommendations Group was that antiviral therapy should only be intended for congenitally infected neonates and premature infants [2,7]. Newborns with asymptomatic or mildly symptomatic CMV infection should not routinely receive antiviral therapy [2,7]. However, controversy remains over antiviral therapy for severe symptomatic CMV infection.

Hu et al. reported that three cases did not respond to intravenous immunoglobulins, but their viral load was significantly reduced following treatment with ganciclovir, followed by an increase in platelet count [2,8,9]. In addition, a case series in Korea found that among immunocompetent children aged one month to 15 years with CMV-related thrombocytopenia, about one-third of patients required antiviral therapy due to severe and refractory thrombocytopenia [10,11]. These reports suggest that antiviral therapy could be considered in cases of CMV-associated thrombocytopenia involving a complex course of the disease and recurrent or refractory thrombocytopenia after treatment with IV immunoglobulin. Regarding our patient, her platelet count recovered with decreasing CMV loads after the start of antiviral therapy.

The use of GCV or other anti-CMV agents has a wide range of potential side effects, such as carcinogenesis, teratogenicity, and bone marrow suppression, and GCV is contraindicated in patients with thrombocytopenia (<25,000/ml) [6,10]. We followed our patient clinically and monitored platelet and liver enzyme levels. Our patient had none of these side effects.

## Conclusions

In conclusion, thrombocytopenia is one of the common clinical manifestations in congenital or infantile symptomatic CMV infection. For immunocompetent children, antiviral therapy might be necessary for CMV thrombocytopenia refractory to standard therapies for ITP. It is useful to report these cases to make recommendations specifying the indications for antiviral treatment in infants with severe CMV infection.
